# An efficient and secure compression technique for data protection using burrows-wheeler transform algorithm

**DOI:** 10.1016/j.heliyon.2023.e17602

**Published:** 2023-06-23

**Authors:** M Baritha Begum, N. Deepa, Mueen Uddin, Rajesh Kaluri, Maha Abdelhaq, Raed Alsaqour

**Affiliations:** a Department of Electronics and Communication Engineering, Saranathan College of Engineering, Tiruchirapalli, 620012, Tamil Nadu, India; b Department of Computer Science, School of Engineering & Technology, Pondicherry University, Karaikal Campus, Karaikal, 609605, Puducherry UT, India; c College of Computing and Information Technology, University of Doha for Science and Technology, Doha, 24449, Qatar; d School of Information Technology and Engineering, Vellore Institute of Technology, India; e Department of Information Technology, College of Computer and Information Sciences, Princess Nourah Bint Abdulrahman University, P.O. Box 84428, Riyadh, 11671, Saudi Arabia; f Department of Information Technology, College of Computing and Informatics, Saudi Electronic University, 93499, Riyadh, Saudi Arabia

**Keywords:** Burrows-wheeler transform, Run-length encoding (RLE), Encryption, Move-to-front transform, Keyed scrambling

## Abstract

Data stored on physical storage devices and transmitted over communication channels often have a lot of redundant information, which can be reduced through compression techniques to conserve space and reduce the time it takes to transmit the data. The need for adequate security measures, such as secret key control in specific techniques, raises concerns about data exposure to potential attacks. Encryption plays a vital role in safeguarding information and maintaining its confidentiality by utilizing a secret key to make the data unreadable and unalterable. The focus of this paper is to tackle the challenge of simultaneously compressing and encrypting data without affecting the efficacy of either process. The authors propose an efficient and secure compression method incorporating a secret key to accomplish this goal. Encoding input data involves scrambling it with a generated key and then transforming it through the Burrows-Wheeler Transform (BWT). Subsequently, the output from the BWT is compressed through both Move-To-Front Transform and Run-Length Encoding. This method blends the cryptographic principles of confusion and diffusion into the compression process, enhancing its performance. The proposed technique is geared towards providing robust encryption and sufficient compression. Experimentation results show that it outperforms other techniques in terms of compression ratio. A security analysis of the technique has determined that it is susceptible to the secret key and plaintext, as measured by the unicity distance. Additionally, the results of the proposed technique showed a significant improvement with a compression ratio close to 90% after passing all the test text files.

## 1 Introduction

Compression refers to the process of representing using the fewest possible bits. This technique reduces the storage needed and increases the data’s ability to transmit. The purpose of reducing redundant information in stored and transmitted data is to improve efficiency, optimize resource utilization, and enhance data management. Redundancy refers to duplicate or repetitive data within a dataset or transmission.

Compression techniques allow data to be stored more compactly, reducing the required storage space. Compressed data requires fewer bits to be transferred during transmission, reducing transmission time and lower bandwidth usage. Compression is crucial in optimizing storage capacity, improving data transfer efficiency, and enabling faster processing of large datasets [1].

By compressing data, the amount of storage space required is reduced. This is particularly advantageous for large datasets, files, or archives. Compression techniques eliminate redundancy, encode patterns more efficiently, and minimize the overall size of the data. This leads to significant space savings on storage devices or servers.

Compression can significantly reduce transmission time when transmitting data over networks or the Internet. Compressed data requires fewer bits to represent the same information, resulting in smaller file sizes. Smaller files can be transmitted more quickly, especially in scenarios with limited bandwidth or when transferring large volumes of data. Faster transmission can improve efficiency, reduce latency, and enhance user experience.

Compression techniques allow data to be stored more compactly, reducing the required storage space. Compressed data requires fewer bits to be transferred during transmission, reducing transmission time and lower bandwidth usage. Compression is crucial in optimizing storage capacity, improving data transfer efficiency, and enabling faster processing of large datasets.

Compression is classified into two categories based on the necessary reconstruction level: lossless and lossy. Lossless compression results in an identical reconstruction of the original data, whereas lossy compression produces reconstructed data that is not the same as the original data. Lossy compression is frequently utilized in internet applications, particularly for streaming media and telephony. Reducing the amount of data needed to represent text is called text compression. This technique is essential for lossless compression, as the reconstructed text must be the same as the original input. Any slight differences in the reconstructed text can result in a different meaning. Text compression can be achieved using various statistical modeling algorithms, including the Burrows-Wheeler Transform [2], Prediction by Partial Matching, Lempel-Ziv-1977, and Lempel-Ziv-Welch. The compression rate can be determined using various algorithms, some of which maintain the original information and are considered lossless.

In contrast, others may cause a loss of specific information during compression. It is essential to be aware that each compression method is specifically designed for a particular type of image and may not provide optimal results for other images. Some algorithms can modify variables to enhance the compression results and produce a more refined image. Encryption safeguards information and maintains its confidentiality by rendering data unreadable to unauthorized parties. It is critical in protecting sensitive information, securing communication, meeting compliance requirements, building trust, and ensuring data integrity. Encryption is essential to modern information security strategies and helps mitigate the risks associated with unauthorized access and data breaches [3].

Secret key control measures are significant in compressed and encrypted data security. They are essential data for maintaining the data's confidentiality and integrability.

Organizations can enhance the security of compressed and encrypted data by implementing robust secret key control measures. These measures protect against unauthorized access to sensitive information, minimize the risk of key compromise, and ensure the confidentiality and integrity of data throughout its lifecycle. Effective key control measures are crucial for encryption systems' overall strength and reliability.

To secure the message, using cryptography before concealing it is advisable. Several cryptography algorithms are widely utilized, including the Rivest-Shamir-Adlemanalgorithm, the Advanced Encryption Standard algorithm, Blowfish, and the Data Encryption Standard [4,5].

Encryption provides privacy and confidentiality for the data. Encryption protects the data against an intruder who cannot read the information. Data compression is a technique that eliminates repeated character strings within a file. This process results in a compressed file with more evenly distributed characters [6]. As a result, the compressed file requires less time for encryption, decryption, and transmission, leading to shorter plaintext and cipher text. The standard encryption algorithm and compression are executed serialized and are widely preferred for confidentiality to compressed data [7]. To illustrate, a collection of files can be bundled in a WinZip archive, each undergoing its own compression and encryption process [8]. The cipher text characters should have an almost uniform distribution produced by a good encryption algorithm. Compression algorithms find and remove repetitive or redundant information within a dataset, resulting in a more miniature data representation. However, encryption algorithms rely on the randomness and unpredictability of the data to ensure its confidentiality and integrity. Encrypting compressed data can lead to a loss of randomness and introduce patterns that may make it easier for an attacker to decrypt the data.

When compressing data, the compression algorithm creates a compressed representation smaller than the original data. This size reduction can reveal information about the structure or content of the data, which could aid an attacker in breaking the encryption. To address this challenge, it is recommended to apply encryption before compression. By encrypting the data first and then compressing the encrypted data, the compression algorithm cannot exploit patterns or redundancies within the data, as it will appear as random data. This approach ensures that the compression process does not compromise the security of the encrypted data.

One effective method for achieving a high degree of secrecy is to use the compress-then-encrypt approach. It is important to note that the mentioned approach has a significant drawback - it could be more flexible when analyzing compressed data for specific patterns [9]. Encryption can make processing compressed data for text matching challenging, restricting potential use cases. Data compression primarily aims to reduce memory space or transmission time, while cryptography secures the data. Encryption and compression are the two main features when the data is transmitted over the Internet or another insecure channel. Encryption provides privacy and confidentiality for the data. The cipher text characters should have an almost uniform distribution produced by a good encryption algorithm. In some instances, performing encryption before compression may be preferable, particularly when transmitting redundant information over a restricted and unsecured communication channel. This approach ensures the confidentiality of the data being transmitted while also conserving bandwidth.

**Fig. 1:**
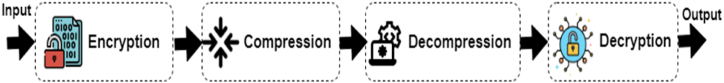
General Block diagram for Encryption before Compression.

The diagram represented in Fig. 1 presents an all-in-one method for combining data compression and encryption in one step. This is considered the second technique for ensuring the security of compressed information. This joint approach addresses two challenges in transmitting confidential data over a network: speed and security. Combining these two processes in a single step efficiently addresses both compression and security needs. Input text scrambling by permutation, Burrows-Wheeler Transform (BWT) [2], Move-to-Front (MTF), and Run-Length Coding can be combined to achieve efficient data compression. When combined, these techniques create a robust compression scheme. First, the text is scrambled to introduce randomness and disrupt patterns. Then, permutation, BWT, MTF, and Run-Length Coding are applied sequentially to reduce the data size further. This combination allows for efficient compression while maintaining the integrity and confidentiality of the data.

An approach to secure and efficient compression is established by utilizing scrambled input. This is achieved by generating a key and applying a permutation. The frequency of the characters in the input text is used as the key value. The proposed method involves scrambling input data with a generated key, transforming it through BWT, and compressing it through Move-To-Front and Run-Length Encoding.

The main contributions are.

1. The proposed work will enhance data security through a combined approach of Encryption and Compression without impacting each other’s performance.
2. An approach to secure and efficient compression is established by utilizing scrambled input. This is achieved by generating a key and applying a permutation. The frequency of the characters in the input text is used as the key value.
3. The proposed method involves scrambling input data with a generated key, transforming it through BWT, and compressing it through Move-To-Front and Run-Length Encoding.
4. The compression process integrates cryptography's confusion and diffusion properties, improving performance by measuring parameter unicity distance.
5. The proposed technique is designed for strong encryption and acceptable compression, balancing security and compression efficiency.

## 2 Literature survey

The challenge of data compression and encryption requires significant computational resources to handle extensive data. Thus, it is challenging to implement them together. There are two ways of combining these processes: encrypting first and compressing first. Encrypting first involves incorporating compression into the encryption process for increased security, but this method results in lower compression ratios and performance than typical compression techniques. On the other hand, compressing first involves incorporating encryption into the compression process, but it is a more complicated and time-consuming approach.

Protecting data during preservation or transmission aims to increase its immunity against various security breaches and prevent unintended changes and unauthorized or illegal access. Numerous efforts have been made to address security challenges in data protection. The research in Ref. [10] highlights the relevance of cryptography in safeguarding data during preservation and transmission. Nonetheless, cryptographic systems have limitations related to computational time and space, as well as their performance and security [11]. As conventional cryptographic methods may not satisfy the needs of contemporary data storage and communication systems [12], scientists are seeking more secure alternatives. Scientists have designed efficient cryptography based on chaos theory and thoroughly evaluated its security features. The unpredictable and delicate qualities of chaotic nonlinear systems are appropriate for cryptographic purposes [13].

Meanwhile, the size of data can be reduced through various data compression methods during preservation and transmission, such as Arithmetic Coding (AC), Huffman Coding (HC), and Lempel-Ziv (LZ) [14,15]. Data compression changes the input data into a smaller, compressed format by removing redundancies. Different compression systems are appropriate for different types of input data and produce different outputs. Kim and colleagues [16] proposed a technique combining compression and security by combining conventional arithmetic coding with the Advanced Encryption Standard (AES). The suggested method employs interval division in arithmetic coding to furnish confidentiality in multimedia files such as video. Although AES is considered secure, its future security cannot be ensured. The proposed technique has the same processing speed as traditional arithmetic coding and adds minimal complexity [17].

Secure Arithmetic Coding (SAC) security relies on interval division through arithmetic coding and permutation. SAC was found to be susceptible to adaptive chosen-ciphertext attacks if the attacker had access to the decoder. To address this issue, a technique incorporating context-based coding offers a better compression ratio, making it easier to integrate with modern standards. In addition, a method for an adaptive chosen-ciphertext attack was proposed, with a runtime O(N) analysis for recovering key vectors. Here, N corresponds to the length of the symbol sequence [18]. A different research investigation was conducted to assess the security of the Lempel-Ziv Welch algorithm. It revealed a chosen-plaintext attack that could undermine the encryption and encoding of single-symbol strings, even if the LZW algorithm was considered secure. Several techniques were implemented to enhance the security of the LZW algorithm, including the insertion of dictionary entries at random and the permutation of entries. However, these methods decreased compression efficiency compared to the original LZW algorithm [19]. To improve the performance of the bzip2 compression pipeline, parallelization of the stages that involve the Burrows-Wheeler Transform (BWT), move-to-front transform (MTF), and Huffman coding can be optimized. This method enhances the efficiency of compression by utilizing the BWT and MTF reverse transformations on the contents of a file, yielding better results in terms of compression. However, it may be slower compared to traditional serial bzip2. The primary goal of secure compression is to convert textual data into a condensed format that can be compressed more efficiently, utilizing the inherent duplicity in language [20]. To achieve this, the Intelligent Dictionary-based Encoding (IDBE) approach is utilized and provides an added layer of security. The algorithm consists of two steps: forming an intelligent dictionary and encoding the input text. This preliminary text processing leads to a noticeable improvement in compression efficiency.

A privacy-preserving approach for text retrieval is implemented by utilizing carefully crafted dummy queries to protect user privacy. The generated dummy queries have similar feature distributions but unrelated topics to the user queries, ensuring effective privacy preservation without compromising the accuracy and usability of the text retrieval system [[21], [22], [23]]. The EMR cloud hierarchical storage model is adopted to ensure the confidentiality of electronic medical records stored on an untrusted cloud. This model involves storing lightweight EMR data items, such as basic patient information, on a local server while encrypting and storing heavy EMR data items, such as medical images, on the cloud. This approach maintains the security and confidentiality of electronic medical records within the cloud environment [[24], [25], [26]].

A comprehensive framework is established to protect user privacy in personalized information retrieval. This framework incorporates generating and submitting well-designed dummy requests alongside user requests to the server. The aim is to obscure user requests and effectively safeguard user privacy. Additionally, the framework includes a privacy model and an accompanying implementation algorithm to enhance the protection of user privacy [[27], [28], [29]].

The research focused on safeguarding users' behavior privacy on commercial websites involves constructing a group of dummy requests on a trusted client. These dummy requests are submitted alongside a user’s commodity viewing request to the untrusted server side, aiming to confuse and conceal user preferences. This research initiative significantly protects user privacy within commercial websites [30,31].

Constructing a set of dummy requests on a trusted client, submitted alongside a user's commodity viewing request to the untrusted server side, represents valuable research in protecting users' behavior privacy on commercial websites. This approach, designed to confuse and conceal user preferences, is significant for developing privacy-preserving e-commerce platforms [32]. A Chinese keyword-based book search service implements careful modification of user query sequences to safeguard user topic privacy on an untrusted server. By obscuring the user query topics, this approach ensures privacy protection while maintaining the accuracy of the book search service. Its positive impact extends to developing privacy-preserving text retrieval platforms, particularly in untrusted network environments [33].

The current work addresses the challenges by combining data compression and encryption into a single process using a secret key. The proposed system starts with a step to generate a key and apply permutation to the input sequence. After permuting the input, it undergoes encoding by the Burrows-Wheeler Transform (BWT) algorithm, which sorts data in blocks instead of sequentially. The resulting output of BWT is then directed to the Move-To-Front Transform encoder and subsequently to the Run Length encoder, which acts as the final coding approach. Combining the cryptographic properties of confusion and diffusion into the compression process enhances performance and overcomes the limitations of previous methods. Cryptographic techniques add an additional layer of security and efficiency by incorporating confusion and diffusion principles into the compression process. Confusion prevents information leakage and protects the data from unauthorized access, while diffusion optimizes the compressed representation by distributing information across the output. These principles work together to enhance the performance of compression algorithms, ensuring that both security and efficiency are achieved in the process.

The subsequent sections of this paper follow the following structure: Section 3 offers a comprehensive explanation of the proposed technique. Section 4 delves into the discussion of the obtained results from the technique. Lastly, Section 5 encompasses concluding remarks on the findings of this proposed method.

## 3 Proposed system

The proposed technique incorporates text scrambling, permutation, Burrows-Wheeler Transform (BWT), Move-to-Front (MTF), and Run-Length Coding to enhance encryption robustness and achieve effective data compression. This amalgamation of techniques offers the potential for efficient compression while ensuring strong encryption measures. When combined, these techniques create a robust compression scheme. First, the text is scrambled to introduce randomness and disrupt patterns. Then, permutation, BWT, MTF, and Run-Length Coding are applied sequentially to reduce the data size further. This combination allows for efficient compression while maintaining the integrity and confidentiality of the data.

By combining these techniques, the above approach ensures that the encrypted data remains secure through robust encryption mechanisms while achieving sufficient compression by leveraging patterns and redundancies within the data. The integration of encryption and compression techniques in a coherent manner enables the achievement of both security and efficiency goals.

• Text scrambling refers to rearranging the characters in a text to make it more challenging to decipher. This technique can introduce randomness and disrupt patterns, enhancing the security of the data.
• Permutation involves rearranging the order of elements in a sequence. The characters' positions are changed by applying permutation to the text, further obfuscating the original message.
• The Burrows-Wheeler Transform (BWT) is a reversible data transformation technique that rearranges characters based on context. It groups similar characters, making the subsequent compression algorithms more effective.
• Move-to-Front (MTF) is a simple but effective technique that reorganizes the character set based on its frequency of occurrence. Frequently occurring characters are placed at the front, enabling efficient encoding during compression.
• Run-Length Coding is a compression method that replaces consecutive repeated characters or sequences with a count and a single occurrence. It significantly reduces the size of repetitive data.

**Fig. 2:**
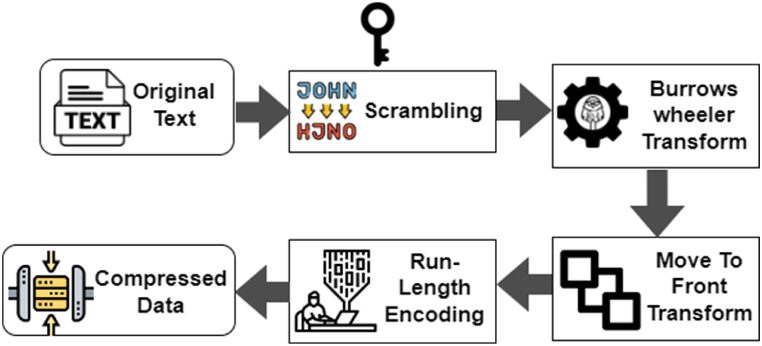
Block diagram of the proposed system Encoder.

The proposed work enhances data security through a combined approach of Encryption and Compression without impacting each other’s performance. An approach to secure and efficient compression is established by utilizing scrambled input. This is achieved by generating a key and applying a permutation. The frequency of the characters in the input text is used as the key value. The proposed method involves scrambling input data with a generated key, transforming it through BWT, and compressing it through Move-To-Front and Run-Length Encoding. The compression process integrates cryptography's confusion and diffusion properties, improving performance by measuring parameter unicity distance. The block diagram of the encoder in the proposed system is shown in Fig. 2, which also illustrates the proposed system's process flow.

The proposed system comprises four steps:

1. A two-step scrambling process enhances the input sequence security before applying the Burrows-Wheeler Transform. The first step involves generating a unique key value for each input of the same size, which is determined by the Letter frequency distribution. This key value is then used to transform the input data into matrix form, and the second step involves applying a column permutation to the matrix. Once the permutation is complete, the data is converted back into vector form for further processing. To determine the Frequency distribution of characters in alphabetical order, it is necessary to compute the key. This key can be squared when the length of the text has an integer square root equal to the key size. This scrambling aims to encrypt the input data, making it more secure for further processing.
2. After the input sequence is scrambled, it is processed by the Burrows-Wheeler Transform (BWT) for improved compression. The BWT block sorting algorithm sorts data blocks to improve compression. The algorithm begins by performing N cyclic right shifts on the scrambled input, followed by a lexicographical sort. From the sorted matrix, the last character of each rotation is obtained as the output of the BWT encoding. The BWT is a lossless block sorting algorithm that sorts data blocks without compressing them. The input for the encoding process using BWT is a scrambled file.
3. The output from the BWT is then passed on to the Move-To-Front Transform encoder. This transformation reduces the entropy of the data but does not lead to compression. The Move-To-Front (MTF) transformation is a data compression technique that rearranges symbols in an input sequence so that the most frequently occurring symbols appear earlier. This approach involves assigning a location to every symbol within the input, and whenever a symbol is encountered, it is relocated to the beginning of the list. As a result, when the same symbol appears again, it is more likely to have a lower position in the list, which can be encoded with fewer bits. The outcome of this encoding is a sequence of integers that indicates the position of the symbols in the list.
4. The Run-Length encoder performs the final stage of the encoding process, which reduces the size of the data sequence. MTF is often used with other coding methods, such as Run-length encoding, to achieve better compression. This simple and efficient technique is easily implementable and can be integrated into a compression system. RLE is a coding scheme in which consecutive repeated values are replaced with a single occurrence and a count. The purpose of RLE is to compress sets that contain long runs of repeating values by representing them as a single value and a count of repetitions.

**Fig. 3:**
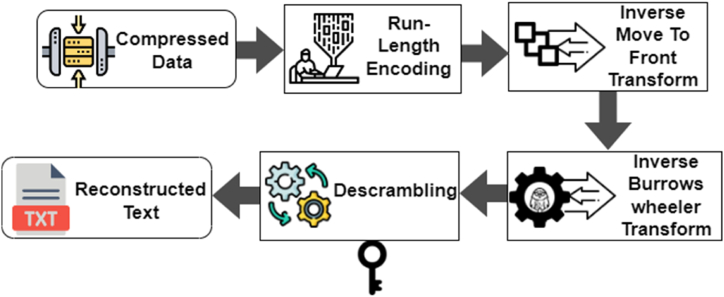
Reconstruction of original text.

The block diagram of the proposed decoder system is illustrated in Fig. 3. The decoding process starts with the binary data obtained from the encoded input. This binary data contains two components: the index of the original string within the sorted table and the value of the final column in the sorted table.

Next, run-length decoding is performed, which reverses the encoding process and reconstructs the original data by replacing the consecutive repeated values with a single occurrence and a count.

After the run-length decoding, the Inverse Move-To-Front Transform is applied. This transformation is the reverse operation of the Move-To-Front Transform used during the encoding process. It rearranges the symbols in the sequence based on their positions in the list.

The output of the Inverse Move-To-Front Transform gives the columns of the Burrows-Wheeler Transform (BWT) matrix. This step helps in reconstructing the scrambled input data.

To reconstruct the original input data, a vector table is created based on the columns of the BWT matrix. This vector table aids in mapping the columns back to their original positions.

Finally, the reconstructed data undergoes an inverse permutation operation using the same key value initially used for scrambling the input data. This operation successfully descrambles the input data, recovering the original sequence.

The block diagram and the description of the decoding process help illustrate how the proposed system reverses the steps of the encoding process to retrieve the original input data.

To determine the compression level, one can calculate the ratio of the compressed output size to the original uncompressed input size. Quantifying the information content of the data involves calculating the bitrate. Several measurements are used to evaluate the effectiveness of the compression technique, including the bitrate in the output data, the entropy of both the input and output data, the unicity distance for different key sizes, and the compression ratio for various input sizes. After compression, the data has fewer bits per character, and its entropy is greater than that of the original input sequence. As the key size increases, the unicity distance also increases, and the compression ratio may fluctuate depending on the input file size.

### 3.1 Input text scrambling

A scrambler is a telecommunications tool that modifies a data stream before transmission by replacing data sequences with different sequences. At the receiving end, a descrambler performs the reverse operation. The number of possible permutations is denoted by “n,” and each permutation is represented by an integer “N” where 0 ≤ N ≤ n! For 32-bit words, the value of “n” is limited to 12, while for 64-bit words, it is limited to 20. The input text is denoted as “T,” and its symbols are represented as “t1, t2, … tn.” The input text can be written as “T = t1, t2, …, tn,” where each “ti” belongs to the alphabet “Σ” with 1 ≤ i ≤ n. The character set used in the input text, “Σ,” is defined as “Σ = {ε1, ε2, …, εk}”.

To generate the key, it is necessary to calculate the frequency distribution of each character in the input sequence and then sort them in lexicographical order. The resulting frequencies of the characters are then used as the key values. If the length of the input text has a square root that is a whole number equal to the size of the key, a square matrix is formed. However, it should be noted that the product of the matrix's rows and columns may not always be equal to the number of elements in the input data, denoted as N. The process of generating the key involves the following steps:

1. First, the input text is sorted in ascending order. Then, the frequency of each symbol present in the input text is determined.
2. The key value used is the frequency of each symbol, and the input data is converted into a matrix by following a specific process. The size of the key determines the number of columns in the matrix, while the number of rows is determined using Eq. (1).
3. The matrix columns undergo shift steps based on their key value.

(1) $$Number\;of\;rows\;{=}^{\prime \prime }Input\;file\;size\;in\;byte^{\prime \prime }/\prime \prime Key\;size\;in\;byte\prime \prime$$

To ensure the validity of the key, each of its elements should be less than or equal to the total number of rows. This can be confirmed by comparing each element in the key with the number of rows. If a key element exceeds the number of rows, it can be recalculated using Eq. (2).

(2) $$New\;key\;value=remainder\;\left(\frac{Key\;value}{Number\;of\;rows}\right)$$

1. It remains unchanged if the key value equals or exceeds the number of rows. Then, the columns of the matrix are reorganized based on the updated key value.
2. The original input data is transformed into a rearranged matrix, then converted into a single vector that changes the data order. For instance, let us consider the input sequence “mississippi”. We calculate the frequency of each character and arrange them into a matrix with 4 columns. The number of rows in the matrix is determined, and a new key is obtained by rearranging the previous key values as [1,1,2,1]. The resulting vector would be “pmipissis@si”, following the expression: ((&miss@&issi@&ppi@)) ([1,1,2,1]) → "pmipissis@si”. The key values determine the order in which the matrix columns should be rearranged. The first value in the key, 1, indicates the downward cyclic shift applied to the leftmost column, and so on. During the descrambling step in the decoding process, the original input text can be recovered by applying a reversible rearrangement.

### 3.2 Burrows-Wheeler Transform

After the input sequence is scrambled, it is processed by the Burrows-Wheeler Transform (BWT) for improved compression. The Burrows-Wheeler Transform (BWT) is a data transformation technique used in data compression algorithms, such as the Burrows-Wheeler Compression (BWC) algorithm. The BWT reorganizes the input data to exploit patterns and redundancy, making it more amenable to compression.

The BWT rearranges the characters in a string based on their circular shifts. This transformation produces a new string where similar characters tend to be grouped, enabling better compression ratios by subsequent compression algorithms.

In the proposed technique of encrypt-then-compress.the Burrows-Wheeler Transform can be applied as the initial compression step. The input data is first subjected to the BWT, reorganizing the **scramble** data and exposing potential redundancies and patterns within the transformed string.

**Fig. 4:**
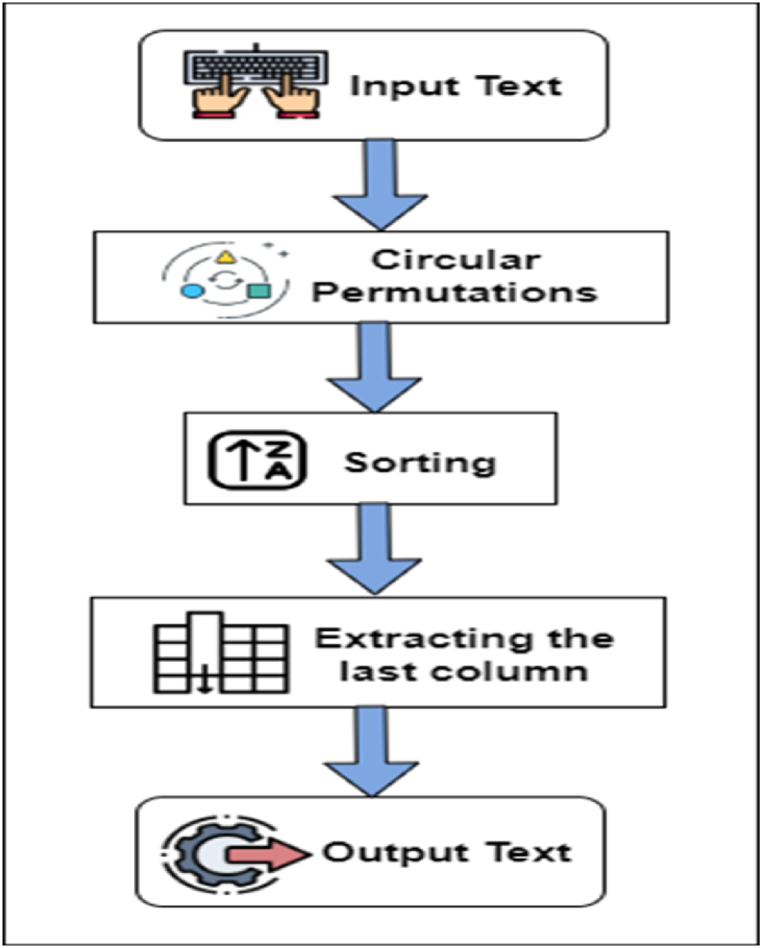
Flowchart of the burrows-wheeler transform.1.**Input:** A scrambled string S of length n, terminated with a unique symbol $ (not present in S). Let L be the column in M containing the terminating $ symbol.2.**Create a cyclic matrix:** To construct a cyclic matrix, we will create a matrix M of size n x n, where each row i represents a cyclic shift of the input string S of length n, starting at the ith symbol of S. Here is the procedure to create the cyclic matrix:a)Initialize an empty matrix M of size n x n.b)Iterate through each row i from 0 to n−1:•Create a cyclic shift of S starting at the ith symbol.•Assign the cyclic shift as the content of row i in matrix M.

The Burrows-Wheeler Transform (BWT) is a data reorganization method used as the initial step in a series of algorithms for data compression. It involves creating a table that contains all cyclic shifts of the scrambled input data and arranging them in lexicographical order. While the data elements remain unchanged, their order is modified in the BWT output. However, the original order of the scrambled data elements can be restored during the reverse operation. It is important to note that the BWT does not compress the data directly but transforms it to make it more amenable to compression by other algorithms. Fig. 4 is the Flowchart of the burrows-wheeler transform.

The equation for the BWT can be represented as Eq. (3)

(3) L[i] = M[i, n-1]

where L[i] is the i-th character of the transformed string L, M is the cyclic matrix of the input string, n is the length of the input string, and M[i, n-1] is the last character of the i-th row in M.

2) Sort the rows of matrix M in lexicographic order based on their contents. After following these steps, the resulting matrix M will be a cyclic matrix, where each row represents a cyclic shift of the input string S. The matrix M will have a size of n x n, where n is the length of the input string S.
3) Extract the last column: Extract the last column of the cyclic matrix M and call it L. This column represents the transformed string after applying BWT. Sort the rows of M lexicographically, considering the last symbol of each row first. If two rows end in the same symbol, compare each row's second to the last symbol, and so on, until all symbols have been considered. The last column of M is the Burrows-Wheeler Transform B(S) of S. This column consists of the last symbol of each row of M, in order of their appearance in the sorted matrix.
4) **Output:** Output the transformed string: Output the transformed string L as the result of BWT. Return B(S).

The input text sequence ‘mississippi’ is transformed into a matrix with the number of columns represented by L (Table 1). The pair of indexes and the final column of the matrix, arranged in lexicographical order, represent the original text sequence. The first row of the matrix, referred to as index 1, contains a copy of the original text. If the input sequence size, N, is 12, a cyclic right shift is performed on the sequence. The resulting 12 sequences are arranged in a lexicographical manner. The encoder transmits a sequence of length 12, created by taking the final letter of each lexicographically sorted and cyclically shifted sequence. The sequence of the last L letters, along with the position of the original sequence in the sorted table, is run-length encoded and sent to the decoder. The transformation produces a pair (L, I) calculated by Eq. (4).

(4) where L is equal to L(I), L(2),…L(N), and the index I is equal to 1.

**Table 1:** Cyclic right shifts and sorted cyclic right shifts.

Index value	Cyclic right shifts	Sorted cyclic right shifts	Index value
1	mississippi$	$mississippi	12
2	ississippi$m	i$mississipp	11
3	ssissippi$mi	ippi$mississ	8
4	sissippi$mis	issippi$miss	5
5	issippi$miss	ississippi$m	2
6	ssippi$missi	mississippi$	1
7	sippi$missis	pi$mississip	10
8	ippi$mississ	ppi$mississi	9
9	ppi$mississi	sippi$missis	7
10	pi$mississip	sissippi$mis	4
11	i$mississipp	ssippi$missi	6
12	$mississippi	ssissippi$mi	3

The BWT procedure is relatively simple and efficient, making it a popular choice for text compression. The inverse transform of BWT can recover the original string from its compressed form.

### 3.3 Move-To-Front Coding

The output from the BWT is then passed on to the Move-To-Front Transform encoder. This transformation reduces the entropy of the data but does not lead to compression. The MTF is a simple transformation that operates on a sequence of symbols, typically characters. It works by maintaining a list or table of symbols in a specific order, such as the order of their appearance or their frequency. When a symbol is encountered in the input data, it is moved to the front of the list/table. This process updates the ordering of symbols based on their recent usage.

The MTF can be useful in compression algorithms, particularly with other techniques such as BWT and Run-Length Encoding (RLE). By rearranging BWT output symbols based on their frequency or usage, the MTF can enhance the compression effectiveness of subsequent compression algorithms.

The move-to-front transformation is a compression method that leverages extended sequences of identical symbols. To execute this technique, it generates a list of N symbols from the source alphabet. Each symbol in the sequence is then assigned a location in the list, and the list is modified by moving the symbol to the front

1. Create a new, empty list to store the items that will be moved to the front.
2. For each item in the original list (BWT output), perform the following steps:
  a. If the item is already in the new list, remove it from its current position and add it to the front of the new list. In move-to-front coding, the symbols in the source alphabet are listed and assigned numbers starting from 0. When a symbol appears for the first time, its position in the list is transmitted and moved to the top of the list.
  b. If the item is not on the new list, add it to the front of the new list.
    1. Once all items in the original list have been processed, the new list will contain the same items as the original list but in a new order that prioritizes frequently accessed items.
    2. Optionally, replace the original list with the new list to make it the active list. Consequently, long sequences of identical symbols are represented by many zeros, while symbols that have not appeared long are assigned larger numbers. This transformation of long runs of identical symbols into many zeros results in a sequence of integers representing the data.
    3. Repeat the above steps whenever an item is accessed in the list.
    4. This method is used in Burrows-Wheeler transform-based compression, applied before entropy coding for improved compression.

### 3.4 Run-length encoding

The Run-Length encoder performs the final stage of the encoding process, which reduces the size of the data sequence. MTF is often used with other coding methods, such as Run-length encoding, to achieve better compression. Run-Length Encoding (RLE) is a simple data compression technique that exploits consecutive repetitions of the same symbol in a data stream. It replaces repeated sequences of symbols with a shorter representation that indicates the symbol and the number of times it repeats consecutively.

In the proposed technique, Run-Length Encoding can be used as one of the subsequent compression algorithms after the initial compression step (such as the Burrows-Wheeler Transform and MTF). Once the data has been transformed or compressed using prior methods, RLE can further reduce the compressed data size.

Run-Length Coding Algorithm:

1. Run length encoding is a data compression method consolidating repetitive data elements into a single value and its count. This technique is especially effective when the data consists of two symbols, and one appears more frequently. This compression method is considered lossless as it does not result in data loss.
2. Create a new, empty list to store the encoded message.
3. Initialize a count variable to zero.
4. For each character in the message, do the following:
  a. Increase the count variable if the current character matches the previous character.
  b. If the current character is not the same as the previous character, append the previous character and the count to the encoded message list, reset the count variable to 1, and update the previous character. In this technique, a sequence of repeating symbols is transformed into a new sequence that includes one or more symbols of the repeating symbol, an escape symbol, and information about the number of repetitions
5. The difference between RLE algorithms can be seen in three aspects - marking the start of a run, the threshold, and encoding the length information. If the length of the repeating symbol is less than the threshold, it remains unchanged; otherwise, it is replaced. Two common methods use a threshold run or an escape symbol when starting a run. In the case of a threshold run, a short sequence of s is employed to define the start of the run, and its length must be equal to or greater than the threshold. On the other hand, an escape symbol $ indicates the beginning of a run, and the run symbol is usually placed after the $ symbol. To ensure that the escape symbol $ is not mistaken for the start of a run, it must be encoded differently. The length information is usually placed directly after the threshold run or $
6. Once all characters in the message have been processed, add the final count and character to the encoded message list.
7. Convert the encoded message list to a string and return the result.
8. Run length encoding is a coding technique that compresses consecutive sequences of identical values in a message by representing them as a single value and its count. Run length encoding aims to compress the data necessary for storage or transmission. This method is widely used in Digital Signal Processing, particularly in image processing and compression applications. Run length encoding can significantly decrease the required data when the original data are identical. In the absence of repeating sequences in the data, using RLE could lead to an increase in the number of values compared to the original data. This could result in data expansion instead of compression.

### 3.5 Run-length decoding algorithm

The run-length decoding is performed, which reverses the encoding process and reconstructs the original data by replacing the consecutive repeated values with a single occurrence and a count.

1. Start with an encoded message you want to decode using the run-length decoding algorithm.
2. Create a new, empty string to store the decoded message.
3. Initialize a count variable to zero.
4. For each character in the encoded message, do the following:
  a. If the current character is a digit, update the count variable by appending the digit to it.
  b. If the current character is a letter, repeat the letter count times, add the resulting string to the decoded message, and reset the count variable to zero.
5. Once all characters in the encoded message have been processed, the decoded message is complete.
6. Moreover, that is it! The run-length coding and decoding algorithm is a simple yet effective technique for compressing and decompressing messages by exploiting the fact that specific sequences of characters repeat frequently.

Despite this, RLE is a lossless compression technique, meaning the exact original data can be obtained after the Run length decoding process.

### 3.6 Move-to-Front (MTF) decoding algorithm

After the run-length decoding, the Inverse Move-To-Front Transform is applied. This transformation is the reverse operation of the Move-To-Front Transform used during the encoding process. It rearranges the symbols in the sequence based on their positions in the list.

The proposed algorithm is utilized to decode a sequence of symbols that have been encoded using the MTF encoding algorithm. The MTF decoding algorithm reverses the operation of the MTF encoding algorithm by reconstructing the original sequence of symbols.

1. Initialize an empty list called “symbol_table” that will hold the symbols used for encoding.
2. Initialize an empty list called “decoded_sequence” to hold the decoded symbols.
3. For each encoded symbol in the input sequence:
  a. Find the index of the symbol in the “symbol_table”.
  b. Remove the symbol from its current position in the “symbol_table”.
  c. Prepend the symbol to the beginning of the “symbol_table”.
  d. Append the symbol to the “decoded_sequence”.
4. Return the “decoded_sequence”.

This procedure will decode an input sequence encoded using the MTF algorithm. The result is the original sequence of symbols that were encoded. The Burrows-Wheeler transform is frequently used with the move-to-front (MTF) technique to enhance the compression rate. The MTF algorithm utilizes two steps: first, it encodes the position of each symbol in the sequence; then, it rearranges the list by placing the symbol at the beginning of the list. This approach is particularly efficient when a symbol repeatedly occurs in the sequence

### 3.7 Burrows-Wheeler Inverse Transform

The output of the Inverse Move-To-Front Transform gives the columns of the Burrows-Wheeler Transform (BWT) matrix. This step helps in reconstructing the scrambled input data.

To reconstruct the original input data, a vector table is created based on the columns of the BWT matrix. This vector table aids in mapping the columns back to their original positions.

The Burrows-Wheeler Transform (BWT) is an algorithm that compresses reversible data. It achieves compression by reorganizing the characters in a string to facilitate the compression process. The BWT can be reversed to recover the original string from its compressed form. To accomplish this, both the first and last columns from the sorted matrix are required. While the BWT does not perform compression, it rearranges the elements to enhance compressibility using techniques like move-to-front encoding. It is important to note that knowledge of the lexicographical ordering used in constructing the BWT of the input data sequence is necessary to search for or reconstruct any portion of the data.

To decode the original input string using the encoded output (L, I) generated by the MTF transform, follow these steps:

1. Obtain the compressed string generated by the MTF transform as the input. Create two columns: “First” and “Last”. The “First” column contains the same characters as the original string but is sorted in lexicographic order. The “Last” column contains the last character of each cyclic permutation of the original string, also sorted in lexicographic order. To decode the original input string of length N using the encoded output (L, I), utilize the F column of the lexicographically sorted matrix, denoted as A. Sort the L string in lexicographic order to calculate the F column. This step helps determine the lexicographic order of the characters in the original input string. To find the last character of the original string, locate the character in the “Last” column corresponding to the first row of the “First” column.
2. To find the character immediately preceding the last character of the original string, locate the character in the “Last” column that is in the same row as the last character of the original string in the “First” column. It is important to note that the “First” and “Last” columns are permutations of the original string. If there is an error in calculating the “First” column, it will be impossible to reconstruct the input file or perform backwards searching. During the decoding process, only the “Last” column, the sorted “First” column, and the index value of the original string in the sorted matrix are available, not the entire sorted matrix.
3. Repeat step 4: Repeat step 4 until you have recovered all the characters of the original string. To find each character, use the current character to locate the preceding character in the “Last” column. The transformation matrix is created by rotating each row of the sorted matrix to the right, resulting in a new matrix labeled A′, calculated by Eq. (5). This applies for each i = 0 to N and each j = 1 to N.

(5) $$A^{\prime }\left[i,j\right]=A[i,\left(j-1\right)modN$$

To obtain the original uncompressed string, reverse the order of the characters in the recovered string after retrieving all the characters of the original string.

The Burrows-Wheeler transform creates a new matrix, A′, based on the original input string. Like the original matrix A, each row in A′ represents a rotated version of the input sequence. The rows in A′ are sorted in lexicographic order, starting from the second character onwards. Consequently, for every row in A, a corresponding row in A′ exists formed by constructing A′ from A.

The relationship between the rows in matrices A and A′ can be determined by utilizing F and L, representing the first columns of matrices A and A′, respectively. This relationship is illustrated in Table 2 and Table 3. Combining these two elements allows a new vector B to be calculated, highlighting the connection between the rows in both matrices.

**Table 2:** Lexicographically sorted matrix.

F	Index value	L	Sorted Matrix	Index value
$	1	i	mississipp	1
I	2	p	$mississip	2
I	3	s	ppi$missis	3
I	4	s	ssippi$mis	4
I	5	m	ississippi$	5
M	6	$	Mississippi	6
P	7	p	i$mississi	7
P	8	i	pi$mississ	8
S	9	s	ippi$missi	9
S	10	s	issippi$mi	10
S	11	i	sippi$miss	11
S	12	i	sissippi$m	12

**Table 3:** Cyclic right shift of the sorted matrix.

Index value	Sorted cyclic right shifts(A)	$A^{\prime }$
1	$mississippi	i$mississipp
2	i$mississipp	pi$mississip
3	ippi$mississ	sippi$missis
4	issippi$miss	sissippi$mis
5	ississippi$m	mississippi$
6	mississippi$	$mississippi
7	pi$mississip	ppi$mississi
8	ppi$mississi	ippi$mississ
9	sippi$missis	ssippi$missi
10	sissippi$mis	ssissippi$mi
11	ssippi$missi	issippi$miss
12	ssissippi$mi	ississippi$m

The vector T can be computed by establishing the relationship between L and F. This is achieved by determining the position of the kth occurrence of a character in L and finding the corresponding position of the kth occurrence of the same character in F. The resulting vector T establishes a one-to-one correspondence between the elements of F and L. In other words, for each element j in L, the corresponding element in F can be identified at index T[j]. For example, if the kth occurrence of a character is L[j], then T[j] will be equal to i, where F[i] represents the kth occurrence of that character. In the provided example, T is represented by (2, 7, 9, 10, 6, 1, 8, 3, 11, 12, 4, 5).

Each row of A needs to be examined to determine the relationship between the characters in matrix A’s first and last columns. For every i within the range of 0 to N-1, the symbols L[i] and F[i] indicate the final and initial characters of the i-th row in matrix A, respectively. As each row in matrix A represents a cyclic permutation of the original input string, the character L[i] appears before the character F[i] in the input string. Eq. (6) can be utilized to express this relationship.

(6) F[T[j]]=L[j]

where T[j]=i,

L[T[j]] Precedes L[j] in the original sequence.

The index I correspond to the position of the original sequence within the sorted matrix. More precisely, the input string is in row I of matrix A. L represents the last character of the original string [I]. The T vector is utilized to determine the characters that precede each character. The original input sequence can be deduced using Eq. (7).

(7) $$i=0,\ldots .N-1:S\left[N-1-i\right]=L\left[T^{i}\left[I\right]\right]$$

$$where\;T^{0}\left[m\right]=m\text{,}$$

$$and\;T^{i+1}\left[m\right]=T\left[T^{i}\left[m\right]\right]$$

Applying the inverse operation of the Burrows-Wheeler transform results in the retrieval of the original string. Subsequently, the reconstructed data undergoes an inverse permutation operation, initially utilizing the same key value employed to scramble the input data. This operation effectively descrambles the input data, restoring the original sequence.

## 4 Results and discussion

The paper presents a novel approach to data compression and encryption that aims to balance reducing storage space and ensuring data security. The approach integrates cryptographic techniques to confuse and diffuse the data, improving performance. After conducting tests on multiple files, the proposed method demonstrated a superior compression ratio compared to other techniques.

However, during the security analysis, it was discovered that the proposed method is susceptible to attacks involving the plaintext and secret key, according to the unicity distance. Future work could address these vulnerabilities while enhancing compression and encryption algorithms.

The evaluation of compression and encryption techniques often involves using benchmarking datasets, such as the Calgary Corpus test files or other widely recognized datasets. These datasets encompass various types of files, including text, images, audio, and more, providing a diverse and representative data set for assessing the performance of compression algorithms.

When evaluating the security of the proposed technique, it is crucial to consider the unicity distance concerning the specific encryption algorithm and key management utilized. The unicity distance is an important security measure, indicating the minimum amount of ciphertext required for an attacker to gain meaningful information about the plaintext.

A larger unicity distance suggests a higher level of security, as it implies that a substantial amount of ciphertext is needed for an attacker to deduce any significant details about the original plaintext. This demonstrates the encryption algorithm's effectiveness and the encryption keys' management. By achieving a larger unicity distance, the proposed technique provides a higher level of protection against unauthorized access and decryption attempts.

### 4.1 Entropy

The entropy H of a random variable X measures the expected level of uncertainty associated with the variable. It can be determined by applying Eq. (8), which considers the probability distribution Px and the alphabet X of the random variable X.

(8) $$H\left(X\right)=-\sum_{x\in X}^{\sum_{X}}P_{X}\left(X\right)\log$$

Information content in a message refers to the minimum number of bits required to express all possible meanings of the message. The entropy of natural language refers to the statistical measurement of how much information, on average, is generated per letter in a given text written in the language. The redundancy property of a natural language is described as the ability to shorten a meaningful message without losing its meaning, and the degree of redundancy in a language quantifies the level of limitations imposed on a text due to its statistical structure.

Due to the dependent nature of successive letters in a language, the entropy is reduced as the relationships among successive letters decrease the entropy. Consequently, the average information content decreases. A language is considered to have a certain degree of redundancy if it can be shown that not all sequences of N letters are equally likely. The entropy test detects encrypted data because the distribution of bytes in the data is uniform, just like the compressed data. When the entropy of the data reaches its maximum value, it indicates that the data is encrypted. The redundancy of a language is calculated as a fraction of excess characters.

$R_{L}$ is a measure of the fraction of excess characters. The redundancy $R_{L}$ is calculated by Eq. (9).

(9) $$R_{L}=1-H_{L}/\log_{2}\left|P\right|$$

where $H_{L}$ = entropy of the plain text

P = size of the message alphabet.

### 4.2 Unicity distance

The unicity distance is a fundamental concept in cryptography that refers to the minimum amount of ciphertext required to determine the corresponding plaintext with high confidence uniquely. It serves as a measure of the security of an encryption algorithm, indicating the point at which an attacker can break the encryption and recover the original message.

In the context of the proposed technique, the unicity distance is important in evaluating the effectiveness of the encryption algorithm employed. If the unicity distance is relatively short, it suggests that an attacker would need only a small amount of ciphertext to deduce the original plaintext. For a secure encryption technique, a sufficiently large unicity distance is desired. This implies that an attacker would need a significantly large amount of ciphertext to have any meaningful chance of determining the original plaintext. The unicity distance is influenced by various factors, including the encryption algorithm’s design, the encryption key’s length, and the level of randomness introduced during the encryption process. Strong encryption algorithms with longer key lengths generally result in larger unicity distances, making it increasingly difficult for attackers to break the encryption and compromise the system’s security.

The unicity distance in cryptography refers to the minimum length of the ciphertext required to break the cipher and determine the encryption key, eliminating the possibility of false keys through a brute force attack. It represents the point where a limitless adversary can uniquely determine the encryption key. A larger unicity distance indicates a more robust cryptographic system. The unicity distance is influenced by the key's unpredictability and the data repetition level. Eq. (10) is commonly used to calculate the unicity distance. As the redundancy of the data approaches zero, the unicity distance approaches infinity, making a trivial cipher unbreakable even with a ciphertext-only attack.

(10) $$U=H\left(K\right)/\left(R_{L}\;\log_{2}\left|P\right|\right)$$

The entropy of a key is determined by its bit size, and increasing the key size leads to an increase in the unicity distance. The size of a key can be quantified using H(K), which represents the amount of information contained in the key. When all keys are equally likely, the entropy H(K) can be calculated as the logarithm of the number of possible keys, as expressed in Eq. (11).

(11) $$H\left(K\right)=\log_{2}\left(K\right)$$

### 4.3 Compression ratio

The compression ratio is a metric that quantifies the extent of data size reduction achieved through compression. It is often expressed as a ratio or percentage. To calculate the compression ratio, the size of the compressed data is divided by the size of the uncompressed data. Eq. (12) is commonly used to calculate the compression ratio.

Letters = ',:?A'abcdefghiklnoprstuvwy

(12) $$Compression\;Ratio=\frac{The\;size\;of\;the\;compressed\;data}{and\;the\;size\;of\;the\;uncompressed\;data}$$

The space savings, the relative reduction in size compared to the uncompressed size, can be calculated from the compression ratio using Eq. (13).

(13) $$Space\;savings=1-\frac{compressed\;size}{uncompressed\;size}$$

### 4.4 Bits per character

The number of bits per character (BPC) is a metric that quantifies the number of bits required to encode a single data character. Following compression, the BPC value decreases, indicating a reduction in the number of bits needed to represent each character. Eq. (14) can be employed to calculate this BPC value.

(14) $$Bits\;per\;character=\frac{compressed\;size}{uncompressed\;size}*8$$

The data compression process begins by scrambling the input data using a key value. The scrambled data is then fed into the Move-To-Front Transform (MTF) encoder. The MTF encoder reorders the elements based on their frequency, which can enhance compression. After the MTF encoding, the data goes through the run-length encoding process. Run-length encoding condenses consecutive repeated elements into a single value followed by the count of repetitions. This step further reduces the size of the data and contributes to compression. However, it is important to note that the run-length encoding process may not achieve compression if the input sequence contains no repeating elements. In such cases, the encoded data may be longer than the original data, resulting in data expansion instead of compression.

### 4.5 Result

**Fig. 5:**
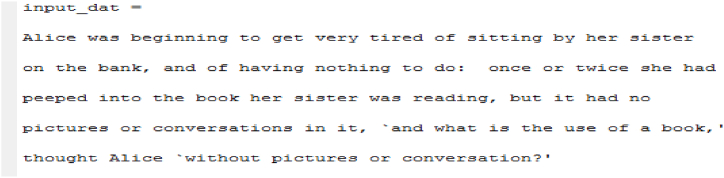
Alphabets in the input data.

Text data is given as the input, as shown in Fig. 5. The size of the input data is given in bytes.

Input file size = 306 bytes.

**Fig. 6:**
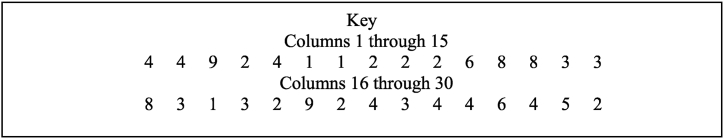
Key value.

The process begins with the input data to generate a key value for scrambling. The key value is determined based on the alphabet used and the distribution of characters in the input sequence, as depicted in Fig. 6. Next, the input data is transformed into a matrix, and a column permutation is applied using the computed key value. This permutation results in the generation of a scrambled input sequence.

**Fig. 7:**
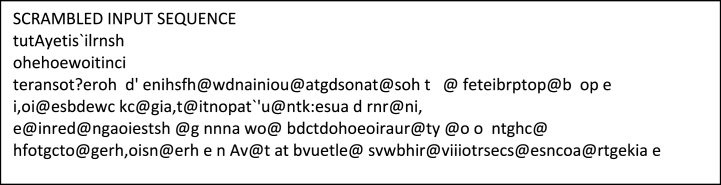
Scrambled input sequcne.

In cases where the frequency of a character's occurrence exceeds the number of rows in the matrix, a new key value is determined by calculating the remainder of the frequency divided by the number of rows. Fig. 7 illustrates this process.

**Fig. 8:**
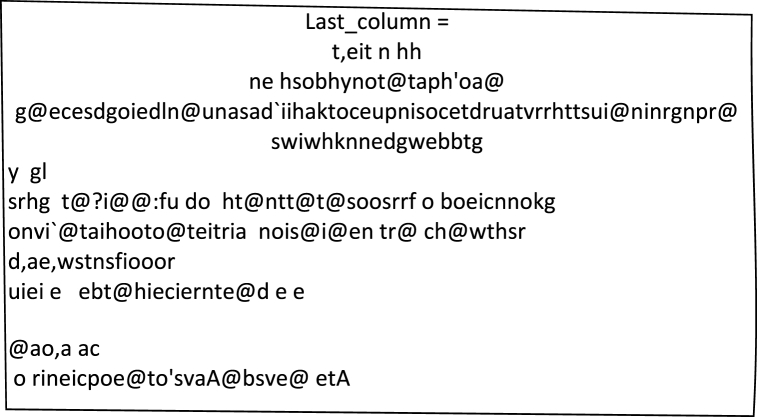
Last column of Burrows-Wheeler Transform Matrix.

The Burrows-Wheeler Transform (BWT) is a technique that rearranges the input data elements to produce encoded data. The resulting encoded data has the same size as the input data. In the BWT, the final column of the transformed matrix is taken as the encoded data, as shown in Fig. 8.

The BWT (Burrows-Wheeler Transform) processes the input data, generating an output. This output is then subjected to the Move-To-Front Transform, which aids in reducing the occurrence of repetitive elements in the data and improving the compression. Subsequently, the transformed data undergoes the Run-Length Encoder, which focuses on compressing repeating sequences within the data to enhance compression efficiency.

Move To Front Transform Input Entropy: 4.2382 bit/symbol

Move To Front Transform output Entropy: 0.9126 bit/symbol

**Fig. 9:**
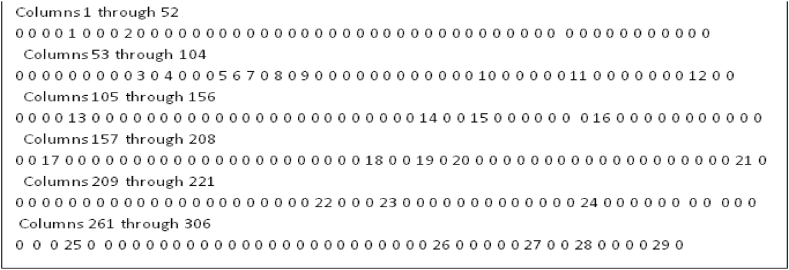
Move-to-Front Transformed data.

**Fig. 10:**
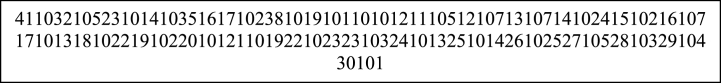
Run-length encoded data.

The Move-to-Front Transformed data is shown in Fig. 9. The run length encoder output is shown in Fig. 10.

The comparison of compression ratios for various data inputs using Keyed Scrambling, BWT, MTF, and RLE is presented in Table 4. It provides information on ten input files labeled TEXT1 to TEXT10, including their input file size, resulting output file size, and the calculated compression ratio.

**Table 4:** Comparison of Compression Ratio of various data inputs.

File Name	Input file size (Bits)	Output file size(Bits)	Compression ratio(Keyed Scrambling + BWT + MTF + RLE)
TEXT1	2448	868	0.4902
TEXT2	6128	402	0.3068
TEXT3	5984	1634	0.3663
TEXT4	8464	1140	0.1815
TEXT5	9976	1298	0.1740
TEXT6	12,312	1368	0.1685
TEXT7	10,736	1732	0.2161
TEXT8	10,992	1316	0.1601
TEXT9	11,480	1556	0.1812
TEXT10	12,856	1384	0.1431

The results indicate that the file TEXT4 achieved the highest compression ratio, with a value of 0.1815. This means the compressed file size was only 0.1815 times the original input file size. Conversely, the file TEXT2 had the worst compression ratio, with a value of 0.3068.

These findings suggest that the effectiveness of the compression method varied for different input files, likely due to the specific data characteristics within each file. On average, the compression ratio achieved by the method was approximately 0.17, indicating that it could significantly reduce the size of the input files. The proposed method demonstrated varying levels of effectiveness across the different input files, with an average compression ratio of around 0.17, indicating substantial file size reduction.

The results of a combined compression and encryption technique, utilizing Keyed Scrambling, BWT, MTF, and RLE, are presented in Table 5. It provides information about ten input files labeled TEXT1 to TEXT10, including their original file size measured in bits. The last column of this table illustrates the ratio of compression and encryption, which is calculated by dividing the compressed and encrypted file size by the original file size.

**Table 5:** Comparison of Bits per Character of various data inputs.

File Name	Input file size (Bits)	Keyed Scrambling + BWT + MTF + RLE
TEXT1	2448	3.9216
TEXT2	6128	2.454
TEXT3	5984	2.930
TEXT4	8464	1.452
TEXT5	9976	1.392
TEXT6	12,312	1.348
TEXT7	10,736	1.728
TEXT8	10,992	1.280
TEXT9	11,480	1.449
TEXT10	12,856	1.144

According to the results, the file named TEXT4 achieved the highest compression and encryption ratio, with a value of 1.452. This indicates that the compressed and encrypted file size was 1.452 times smaller than the original input file size. Conversely, the file TEXT1 demonstrated the worst compression and encryption results, with a ratio of 3.9216. This implies that the effectiveness of the compression and encryption method varied for different types of data within each file. Table 5 presents the results of the combined compression and encryption technique for ten input files. The data shows varying compression and encryption ratios, indicating that the method's effectiveness can vary depending on the specific file and its contents.

The Calgary Corpus is a collection of text and binary data files commonly used for evaluating data compression techniques. The dataset comprises various documents, including Bib, Progc, Paper1, Progl, Progp, and News. Table 6 presents the names, sizes in bytes, and descriptions of these files. The files in the dataset serve different purposes, such as the Bib file, which contains a bibliography. The Paper1 file comprises technical papers, while the Progc file contains source code written in the “C” programming language. The Progl file includes Lisp source code, and the Progp file consists of Pascal source code. Lastly, there is the News file, which contains news-related content. The sizes of these files vary, with the Bib file being the largest at 111,261 bytes and the Progc file being the smallest at 39,611 bytes. Table 6 provides information about several files used in the paper, including their names, sizes in bytes, and brief descriptions.

**Table 6:** Comparison of the compression ratio of various data files.

File Name	Input file size (Bytes)	Existing method		Proposed method	
		Output file size(Bytes)	Compression ratio	Output file size(Bytes)	Compression ratio
Bib	111,261	56,450	0.507366	20841.52	0.18732
Progc	39,611	20,556	0.518947	10801.92	0.2727
Progl	71,646	30,510	0.425844	13225.85	0.1846
Progp	49,379	21,534	0.436096	12576.83	0.2547
Paper1	53,161	28,636	0.538666	10281.34	0.1934
Paper 2	82,199	45,891	0.558291	13365.56	0.1626
News	377,109	39,795	0.105527	97444.97	0.2584

Table 6 compares the existing method [11] of file compression with a proposed method for six different files of varying sizes and types. Table 6 includes the input file size in bytes, the output file size in bytes, and the compression ratio, calculated by dividing the input file size by the output file size.

For all files except “News,” the proposed method yields a significantly smaller output file size and a higher compression ratio than the existing method. This indicates that the proposed method is more effective in reducing file size and achieving better compression. However, in the case of the “News” file, the proposed method results in a much larger output file size. Despite this, the proposed method maintains a higher compression ratio than the existing one.

Overall, the results demonstrate that the proposed method shows promise in reducing file size and improving compression ratios for various file types and sizes. It proves to be more efficient than the existing method, except in the specific case of the “News” file, where further optimization may be required.

**Fig. 11:**
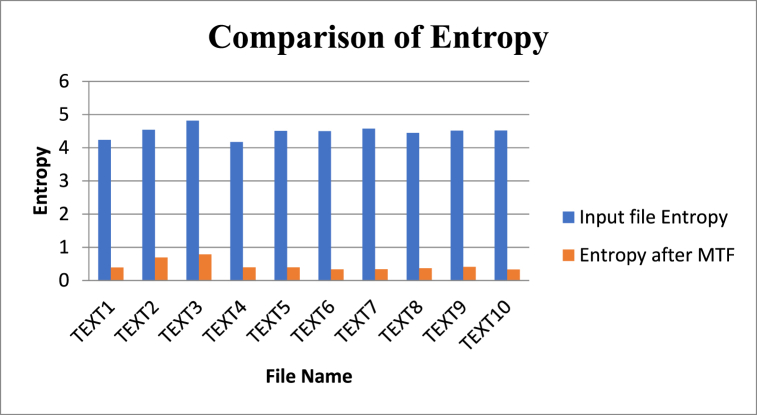
Comparison of entropy of various inputs and MTF Transformed Data.

Table 7 and Fig. 11 present information regarding the input file size (in bits), the input data’s entropy, and the MTF-transformed data for ten text files named TEXT1 to TEXT10. The entropy of the input data measures the level of randomness or unpredictability in the data, with higher values indicating greater randomness. In contrast, the MTF-transformed data's entropy measures the data’s randomness after applying the Move-to-front (MTF) coding technique. The table reveals that the input file size varies from 2448 bits (for TEXT1) to 12,856 bits (for TEXT10). The entropy of the input data ranges from 4.176 (for TEXT4) to 4.812 (for TEXT3). On the other hand, the entropy of the MTF-transformed data ranges from 0.326 (for TEXT10) to 0.7878 (for TEXT3). The application of MTF coding reduces the entropy of the data, making it more compressible.

**Table 7:** Comparison of Entropy of various data inputs and Entropy of MTF Transformed data.

File Name	Input file size (Bits)	Entropy of input data	Entropy of MTF Transformed data
TEXT1	2448	4.238	0.3872
TEXT2	6128	4.539	0.6903
TEXT3	5984	4.812	0.7878
TEXT4	8464	4.176	0.3960
TEXT5	9976	4.5085	0.3941
TEXT6	12,312	4.503	0.3338
TEXT7	10,736	4.576	0.339
TEXT8	10,992	4.4496	0.3703
TEXT9	11,480	4.516	0.411
TEXT10	12,856	4.5217	0.326

The entropy serves as a measure of the complexity or randomness of a message or dataset. Comparing Table 7 and Fig. 11, it is evident that the entropy of the input file is higher than the entropy of the MTF-transformed data. Table 7 provides information about various files and their entropy-related properties. The “File Name” column lists the names of the files, while the “Input file size (Bytes)" column indicates the size of each file in bytes. The “Entropy of input file” column displays the entropy of each file before any coding or compression, and the “Entropy after MTF coding” column shows the entropy of each file after it has undergone MTF coding. Generally, the entropy decreases after applying MTF coding, indicating that the coding method effectively compresses the files by reducing redundancy. The entropy measurement reflects the amount of information contained within the file.

**Fig. 12:**
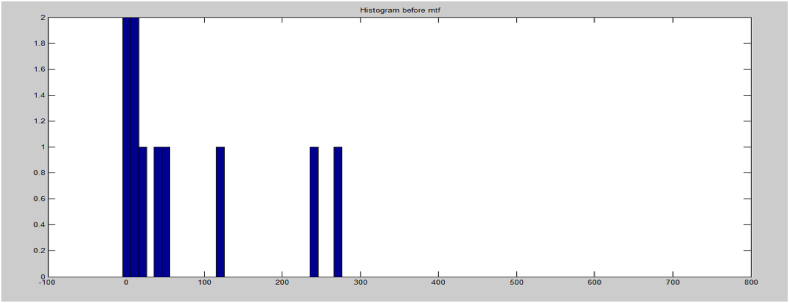
Histogram before MTF.

**Fig. 13:**
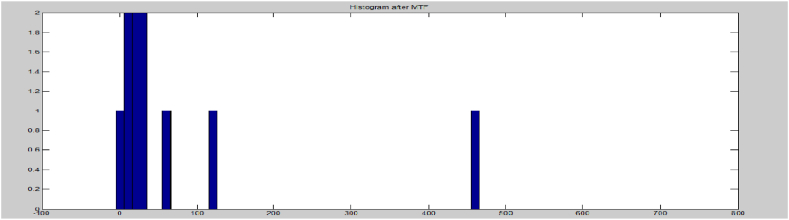
Histogram after MTF.

The protection provided by encryption against cryptanalysis is inversely related to the amount of redundancy present in the message. If there is no uncertainty, the entropy will be zero. Encryption aims to scramble the original message, intentionally increasing its entropy. A well-designed cipher should distribute information from the plaintext over the entire ciphertext. To compute the Shannon entropy of a message, it is possible to use the probability distribution of each character in both the input text and the data that has undergone MTF transformation. Fig. 12, Fig. 13 depict histograms of the data before and after MTF coding, demonstrating that the redundancy of the data decreases after applying Move-To-Front coding. Consequently, the entropy coding technique, such as MTF, can provide greater compression for larger data.

Sequences generated by the Burrows-Wheeler Transform exhibit local frequency correlation. The Move-To-Front transform capitalizes on this correlation to lower the entropy of the message.

Table 8 displays the input file size, key size, and unicity distance for ten text files (TEXT1 to TEXT10). The input file size is measured in bits, while the key size represents the length of the encryption key. The unicity distance indicates the number of possible plaintexts given a ciphertext and a known statistical distribution of the plaintexts. A higher unicity distance corresponds to a more secure encryption system. Table 8 reveals significant variations in the unicity distance among the different text files, ranging from 7.3473 to 8.3067. Similarly, the key size varies from 30 to 57 bits, and the input file size varies from 2448 to 12,856 bits.

**Table 8:** Unicity distance for various data inputs having a different key size.

File Name	Input file size (Bits)	Key size	Unicity distance
TEXT1	2448	30	7.6176
TEXT2	6128	50	7.4054
TEXT3	5984	57	8.3067
TEXT4	8464	37	7.6607
TEXT5	9976	43	8.0404
TEXT6	12,312	43	8.0679
TEXT7	10,736	57	7.39
TEXT8	10,992	43	8.0408
TEXT9	11,480	50	7.3473
TEXT10	12,856	44	8.1939

The unicity distance, which serves as a data security measure, increases with larger key sizes. Typically, a higher unicity distance, ranging from 7 to 8.3 characters, is observed when the key size falls within the range of 30–57 bytes, indicating a stronger cryptosystem. The unicity distance is directly proportional to the key uncertainty and inversely proportional to the redundancy of the input file. A longer unicity distance signifies a more robust cryptographic system. The unicity distance for a specific key size is a valuable metric for assessing the strength of the cryptographic system. As key sizes increase, the unicity distance also increases, resulting in a more secure cryptographic system for input files of the same size.

**Fig. 14:**
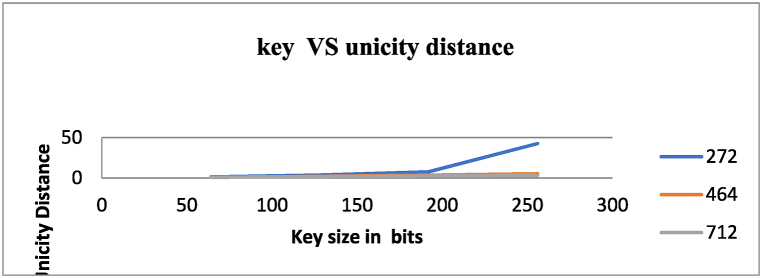
Comparison of unicity distance for different key sizes.

Table 9 and Fig. 14 present data on the unicity distance of different key sizes for three input file sizes in bits: 272, 464, and 712. The key sizes considered are 256 bits, 192 bits, 128 bits, and 64 bits.

**Table 9:** Comparison of unicity distance for different key sizes.

Unicity distance			
Key size	Input file size (bits)		
	272	464	712
64 bits	1.4322	1.0488	0.867
128 bits	3.62	2.11	1.62
192 bits	7.7944	3.497	2.61
256 bits	42.5	5.37	3.126

Unicity distance is a measure of the information entropy in a ciphertext and is used to determine the security of a cipher. A higher unicity distance indicates higher security, as it would require more ciphertext to determine the corresponding plaintext.

For a key size of 64 bits, the unicity distance ranges from 1.0488 for an input file size of 464 bits to 1.4322 for an input file size of 272 bits. This suggests that the cipher's security is relatively low for these input file sizes. The unicity distance increases for key sizes of 128 bits and 192 bits, indicating a higher level of security. For a key size of 128 bits, the unicity distance ranges from 1.62 for an input file size of 712 bits to 2.11 for an input file size of 464 bits. For a key size of 192 bits, the unicity distance ranges from 2.61 for an input file size of 712 bits to 3.497 for an input file size of 464 bits.

For a key size of 256 bits, the unicity distance is significantly higher than for the other key sizes, with a range of 3.126 for an input file size of 712 bits to 42.5 for an input file size of 272 bits. This suggests that the cipher is highly secure for these input file sizes.

Overall, Table 9 provides information on the relationship between key size, input file size, and unicity distance, which measures the cipher’s security. Based on the results, increasing the key size enhances security. Among the tested input file sizes, a key size of 256 bits offers the highest level of security.

## 5 Conclusion

The proposed method offers both data security and compression. The extent of compression achieved depends on the file size and the occurrence of repetitive sequences in the input data. Smaller files tend to result in less compression compared to larger files. The security of the data is determined based on the concept of unicity distance, which is influenced by the redundancy of the input file and the size of the encryption key.

The proposed technique has the potential to undergo evolution or modifications due to technological advancements and the emergence of new security threats. These changes may encompass the following aspects: Hybrid Compression and Encryption Approaches, Machine Learning and Artificial Intelligence integration, Enhanced Encryption Algorithms, Quantum-Safe Encryption implementation, considerations for IoT and Edge Computing, privacy-preserving techniques, and continuous security monitoring.

Compression algorithms typically exploit data redundancies and patterns. However, even a minor change in the data can result in a significant impact on compression effectiveness. If the proposed technique relies heavily on specific data characteristics, it may be less robust against changes in the input data, leading to potential limitations in compression performance. However, a drawback of the proposed method is its vulnerability to attacks involving the secret key and plaintext.

Encrpt-then-Compress technique applications are secure file transfer protocols, Cloud storage and backup solutions, Messaging, and communication platforms, Internet of Things (IoT) devices and sensor data, Financial services, and banking, Healthcare, and telemedicine. Investigate the technique’s applicability to real-world scenarios and address practical considerations such as compatibility with different file formats, support for streaming data, and interoperability with existing systems and tools. Future work could address these vulnerabilities while improving the compression and encryption algorithms. In conclusion, the proposed technique balances strong encryption and acceptable compression, making it suitable for data archival and communication platforms.

## Author contribution statement

M Baritha Begum, N Deepa: Performed the experiments; Conceived and designed the experiments; Wrote the paper.

Mueen Uddin, Rajesh Kaluri: Analyzed and interpreted the data; Contributed reagents, materials, analysis tools or data; Wrote the paper.

Maha Abdelhaq, Raed Alsaqour: Contributed reagents, materials, analysis tools or data.

## Data availability statement

Data will be made available upon request.

## Funding

This research was supported by Princess Nourah bint Abdulrahman University Researchers Supporting Project Number (PNURSP2023R97), Princess Nourah bint Abdulrahman University, Riyadh, Saudi Arabia.

## Declaration of competing interest

The authors declare that they have no known competing financial interests or personal relationships that could have appeared to influence the work reported in this paper

## References

[1] Moffat A., Petri M. (2020). Large-alphabet semi-static entropy coding via asymmetric numeral systems. ACM Trans. Inf. Syst..

[2] Li B., Zhou X., Ning Z., Guan X., Yiu K.C. (2022). Dynamic event-triggered security control for networked control systems with cyber-attacks: a model predictive control approach. Inf. Sci..

[3] Bruno Carpentieri (2018).

[4] Cao K., Wang B., Ding H., Lv L., Dong R., Cheng T. (2021). Improving physical layer security of uplink NOMA via energy harvesting jammers. IEEE Trans. Inf. Forensics Secur..

[5] Chai X.Zheng, Gan Z., Han D., Chen Y. (2018). An image encryption algorithm based on chaotic system and compressive sensing. Signal Process..

[6] Sharma Dr Mukesh, Gandhi smiley (2012). Compression and encryption: an integrated approach. Int. J. Eng. Res. Technol..

[7] Kim Hyungjin, Wen Jiangtao, Villasenor John D. (2007). Secure arithmetic coding. IEEE Trans. Signal Process..

[8] Jiang H., Wang M., Zhao P., Xiao Z., Dustdar S. (2021). A utility-aware general framework with quantifiable privacy preservation for destination prediction in LBSs. IEEE/ACM Trans. Netw..

[9] Zhou Jianto, Au Oscar C., Peter Hon_Wah (2009). Adaptive chosen cipher text attack on secure arithmetic coding. IEEE Trans. Signal Process..

[10] Lee Lung-Jen, Tseng Wang-Dauh, Rung-Bin, Chang Cheng-Ho (2012). 2^n^ Pattern run-length for test data compression. IEEE Trans. Comput. Aided Des. Integrated Circ. Syst..

[11] Lv Z., Chen D., Lou R., Song H. (2020). Industrial security solution for virtual reality. IEEE Internet Things J..

[12] Lv Z., Qiao L., Hossain M.S., Choi B.J. (2021). Analysis of using blockchain to protect the privacy of drone big data. IEEE Network.

[13] Burrows M., Wheeler D.J. (1994).

[14] Hosseini M., Pratas D., Pinho A.J., Cryfa (2019). A secure encryption tool for genomic data. Bioinformatics.

[15] Sardaraz M., Tahir M., Ikram A.A. (2014). SeqCompress: an algorithm for biological sequence compression. Genomics.

[16] Ma J., Hu J. (2022). Safe consensus control of cooperative-competitive multi-agent systems via differential privacy. Kybernetika.

[17] Paul Manas, Mandal Jyotsna Kumar (2012). A general session based bit level block encoding technique using symmetric key cryptography to enhance the security of network-based transmission. Int. J. Comput. Sci., Eng. Inf. Technol. (IJCSEIT).

[18] Morgan Ledwon, Cockburn Bruce F., Han Jie (2020). High-throughput FPGA-based hardware accelerators for deflate compression and decompression using high-level synthesis. IEEE Access.

[19] Sardaraz Muhammad, Tahir Muhammad (2021). SCA-NGS: secure compression algorithm for next generation sequencing data using genetic operators and block sorting. SAGE.

[20] Usama Muhammad, Malluhi Qutaibah M., Zakaria Nordin, Razzak Imran, Iqbal Waheed (2021).

[21] Chanil P., Kwangil A., Paeksan J. (2019). A novel bit-level color image encryption using improved 1D chaotic map. Multimed. Tool. Appl..

[22] Lan R., He J., Wang S., Liu Y., Luo X. (2019). A parameter-selection-based chaotic system. IEEE Trans. Circuits Syst. II: Express Briefs.

[23] Camtepe S., Duda J., Mahboubi A., Morawiecki P., Nepal S., Pawłowski M., Pieprzyk J. (2021). Compcrypt - lightweight ANS-based compression and encryption. IEEE Trans. Inf. Forensics Secur..

[24] Deorowicz S. (2020). FQSqueezer, k-mer-based compression of sequencing data. Sci. Rep..

[25] Camtepe Seyit, Duda Jarek, Mahboubi Arash, Morawiecki Paweł, Nepal Surya (2022). Marcin pawłowski & josef pieprzyk ANS-based compression and encryption with 128-bit security. Int. J. Inf. Secur..

[26] Fan W., Dai W., Li Y. (2017).

[27] Zhang J., Peng S., Gao Y., Zhang Z., Hong Q. (2023). APMSA: adversarial perturbation against model stealing attacks. IEEE Trans. Inf. Forensics Secur..

[28] Wu Zongda, Shen Shigen, Li Huxiong, Zhou Haiping, Lu Chenglang (2021). A basic framework for privacy protection in personalized information retrieval. J. Organ. End User Comput. (JOEUC).

[29] Wu Zongda, Shen Shigen, Zhou Haiping, Li Huxiong, Lu Chenglang (2021). Dongdong zou an effective approach for the protection of user commodity viewing privacy in E-commerce website. Knowl. Base Syst..

[30] Wu Zongda, ShigenShen, ChongzeLin, GuandongXu, Chen Enhong (2023). A confusion method for the protection of user topic privacy in Chinese keyword based book retrieval. ACM Trans. Asian Low-Resour. Lang. Inf. Process..

[31] Wu Zongda, Li Guiling, Shen Shigen, Lian Xinze, Chen Enhong, Xu Guandong (2021). Constructing dummy query sequences to protect location privacy and query privacy in location-based services. World Wide Web.

[32] Wu Zongda, Shen Shigen, Lian Xinze, Su Xinning, Chen Enhong (2020). A dummy-based user privacy protection approach for text information retrieval. Knowl. Base Syst..

[33] Zongda Wu A., Shaolong Xuan A., Jian Xie A., Chongze Lin B., Lu Chenglang (2022). How to ensure the confidentiality of electronic medical records on the cloud: a technical perspective. Comput. Biol. Med..

